# Enhancing Clinical Skills Through Virtual Reality: A Study on 12-Lead Electrocardiogram Placement in Medical Education

**DOI:** 10.7759/cureus.74179

**Published:** 2024-11-21

**Authors:** Kunmilayo Olayeye, Elizabeth (Liz) Oviawe

**Affiliations:** 1 School of Medicine, Nova Southeastern University Dr. Kiran C. Patel College of Osteopathic Medicine, Fort Lauderdale, USA; 2 Division of Institutional Technology, Nova Southeastern University Dr. Kiran C. Patel College of Osteopathic Medicine, Fort Lauderdale, USA

**Keywords:** 12-lead ecg, augmented reality (ar), clinical simulation, immersive learning technology, immersive technology, immersive virtual learning environment (ivle), osteopathic medicine student education, pre-clinical students, simulation in medical education, virtual reality (vr)

## Abstract

Background

Virtual reality (VR) is typically used for entertainment or gaming, but many studies have shown that the applications of VR can also extend to medical and clinical education. This is because VR can help health professionals learn complex subjects, improve memory, and increase interest in abstract concepts. In the context of medical education, the immersive nature of a VR setting allows students and clinicians in training to interact with virtual patients and anatomical structures in a three-dimensional environment or from a clinician's point of view. The benefits of VR also include its ability to allow students to practice clinical skills repeatedly at their own pace and make mistakes without negative consequences to real-life patients.

Objective

This study aims to investigate how VR is beneficial for students learning clinical skills. We used a Likert scale survey to measure the participants’ perception of VR and assess which part of the VR experience was most advantageous to students. Specifically, this study aimed to evaluate whether the Acadicus (Arch Virtual, Madison, WI, US) 12-lead placement VR program offered education benefits to students and whether the tool is something they would consider using in the future for learning.

Methods

Seventy-five first-year osteopathic medical students participated in this study. After completing a 45-minute immersive VR session using the Acadicus program to learn how to place electrocardiogram (ECG) leads on a patient, the students were surveyed with a 23-item Likert scale questionnaire. The survey assessed whether the students had ever used VR or augmented reality (AR) for education or entertainment and their preferred learning styles. Finally, it surveyed their opinions on how they felt the VR experience enhanced their learning and whether this tool was useful academically.

Results

The survey results highlight a generally positive impact on students' understanding and engagement with the 12-lead ECG placement. The highest-scored aspects of the VR experience were its ability to engage users (mean score: 3.77) and the kinesthetic/tactile aspects of the experience (mean score: 3.71). However, the VR experience scored lower in the areas that indicated that the tool helped students understand the spatial and procedural nuances of ECG lead placement, with mean scores of 3.43 for understanding “how” to place leads and 3.38 for understanding “where” leads should be placed.

Conclusions

The findings indicated that most participants found that the VR tool (Acadicus) effectively enhanced their understanding and engagement with the clinical procedure. This study recognized the positive impact that VR technology had on students' confidence levels in the clinical skill of 12-lead ECG placement. Furthermore, based on the data collected, VR is beneficial in medical education and should be integrated into the curriculum of more medical schools due to its ability to enhance student engagement, cater to diverse learning styles, and provide a safe environment for repeated practice of clinical skills. However, there is still a need for continued research regarding VR simulations for clinical skills and with larger groups of participants from different medical institutions. Future research should explore advancements such as haptic feedback integration and gesture tracking necessary for making VR as reflective of reality as possible in the use of VR in medical training.

## Introduction

Virtual reality (VR) is becoming a well-established tool for medical education because it allows users to gain practical skills and develop post-interventional knowledge that can be applied to clinical practice [1]. At Nova Southeastern University Dr. Kiran C. Patel College of Osteopathic Medicine (NSU-KPCOM), VR has become integrated into the medical school’s pre-clinical education to help students gain practical experience in various clinical skills and procedures such as 12-lead electrocardiogram (ECG) placement, Foley catheter placement, and wound stapling, before starting clinical rotations. VR allows students to develop confidence in performing clinical skills in a low-stress environment without compromising patient safety [2,3]. The medical students enrolled in NSU-KPCOM are required to complete five Medical Procedures courses during the first two years of pre-clinical education. These courses are designed to engage the students' clinical thinking abilities and applications of basic clinical skills. Digital learning methods have shown promise in enhancing medical school education, as they provide a more engaging and interactive learning environment for students [4-7].

VR provides users with a three-dimensional sensory-rich immersive experience by engaging visual, auditory, and haptic stimuli [8,9]. As a result, studies show that VR enhances students' motivation to learn in an educational setting because VR allows trainees to observe procedures from a 360-degree view or the surgeon's perspective, which has been shown to increase engagement and provide a more compelling learning experience [10,11]. Despite their conventional association with entertainment, their immersive attributes are relevant in medical education, as they promote active learning and engagement, help students grasp complex anatomy, aid in procedural planning and execution, and provide low-risk practice opportunities [10,12,13]. As a result, VR plays an integral role in medical education, from medical school training to residency and clinical practice [10,12]. Additionally, students of different learning styles require different modalities to better understand academic topics and clinical procedures, so VR’s multisensory immersion has applications for students who benefit from various learning styles [14].

## Materials and methods

Objective

**Figure 1:**
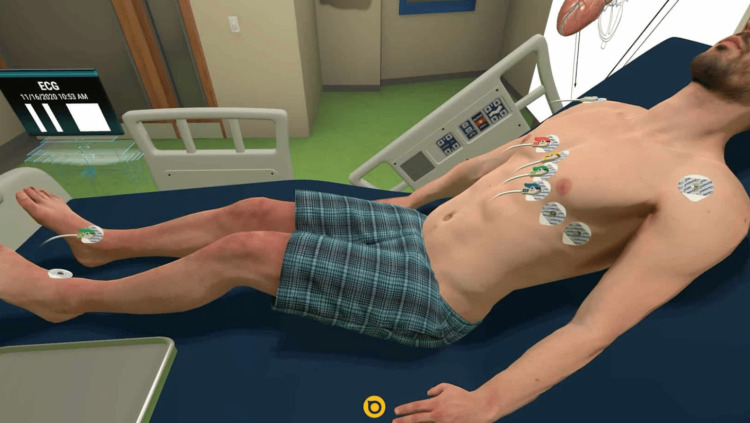
Acadicus electrocardiogram (ECG) simulator Source: Reference [15]

This study aims to investigate how VR may benefit students by evaluating their opinions of the educational benefits of the Acadicus (Arch Virtual, Madison, WI, US) 12-lead placement VR program, as seen in Figure 1. Figure 1 illustrates the placement of the ECG leads. V1, in red, is positioned at the right sternal border at the fourth intercostal space. V2, in yellow, is placed at the left sternal border at the fourth intercostal space. V3, in green, is positioned midway between V2 and V4. V4, in blue, is located at the fifth intercostal space at the midclavicular line. V5, next to V4, is placed at the anterior axillary line at the same horizontal level as V4. V6, next to V5, is positioned at the midaxillary line at the same horizontal level as V4. Lastly, leads right arm (RA), left arm (LA), right leg (RL), and left leg (LL) are placed on their respective limbs. Specifically, the survey used in this study asked various questions to assess which part of the VR experience was most advantageous to students while learning where to place the ECG leads, and whether they would consider using VR in the future for learning.

Hypothesis

VR’s ability to engage multiple senses in a three-dimensional environment is practical for students to develop clinical skills. The hypothesis for this study was tested by evaluating whether students agreed or disagreed with the statements posed on the survey. The questions in the survey used a Likert scale for convenience. Furthermore, using a Likert scale allows the study to be analyzed numerically and provides insights into the potential utility of VR technology in medical education based on the participants' opinions.

Methods

This study involved 75 first-year osteopathic medical students from NSU-KPCOM's Fort Lauderdale campus. To be included in this study, students had to be first-year osteopathic medical students enrolled at NSU KPCOM's Fort Lauderdale campus. Students were respectfully recruited without coercion. All first-year osteopathic medical students at NSU-KPCOM participated in the mandatory Acadicus ECG 12-lead VR experience as part of their Medical Procedures course. However, the survey was voluntary, and each student was approached before the session began. All students received a recruitment flyer along with verbal information about the study. Students were informed that their decision to participate in the study would have no impact on their grades for the course or their standing in the medical school program. Students were also informed that the survey was anonymous and they could withdraw at any time. Consent was obtained during a face-to-face encounter, ensuring that participants were fully informed about the study’s purpose and their rights. Of the students who completed the VR experience, 79 agreed to participate in the study. However, four of the 79 responses were excluded because of their incomplete survey responses. So, for this study, 75 responses were used for analysis (N = 75).

This study received approval from the Nova Southeastern University Institutional Review Board (NSU IRB), under Protocol Number 2023-296. Ethical approval was granted, and all participants provided informed consent before participation.

The VR experience used the Oculus Rift S headsets (Meta Platforms Inc., Menlo Park, CA, US) for the Acadicus 12-lead ECG placement immersive stimulation and lasted about 45 minutes, and the survey on REDCap took participants about five minutes. The participants completed the survey questions after the VR experience was finished. During the immersive VR session, students utilized the Acadicus program, which provided the students with hands-on experience in placing the ECG leads onto a patient in the VR setting. This VR experience integrated a tutorial for the students to watch before completing the ECG lead placement, and there were some questions for the students to complete regarding their learning in the VR stimulation in addition to the procedure itself. Finally, after completing the VR experience, the 75 consenting participants completed the survey on REDCap. The survey had 23 questions and used Likert rating scales. The questions surveyed whether the students had ever used virtual or augmented reality (AR) in the past; it surveyed whether their VR or AR use was for education or entertainment and their preferred learning styles; finally, it surveyed their opinions on how they felt the VR experience enhanced their learning and whether this was a tool they found to be useful academically (Table 1).

**Table 1:** Survey questions VR: virtual reality; AR: augmented reality; NSU-KPCOM: Nova Southeastern University Dr. Kiran C. Patel College of Osteopathic Medicine; ECG: electrocardiogram

Questions and Likert statements	Answer choices
How often have you used virtual reality?	Never, 1-2 times, 3-5 times, 6-10 times, 10+ times
How often have you used augmented reality?	Never, 1-2 times, 3-5 times, 6-10 times, 10+ times
I've used VR/AR for education in the past.	Yes, no
I've used VR/AR for entertainment in the past.	Yes, no
I've used VR/AR for education and entertainment in the past.	Yes, no
I've used VR/AR for neither education nor entertainment.	Yes, no
I am a visual learner.	Strongly disagree, disagree, neutral, agree, strongly agree
I am an aural learner.	Strongly disagree, disagree, neutral, agree, strongly agree
I am a reading/writing learner.	Strongly disagree, disagree, neutral, agree, strongly agree
I am a kinesthetic learner.	Strongly disagree, disagree, neutral, agree, strongly agree
Supplemental VR tools would have been beneficial in the NSU-KPCOM’s Cardiology course.	Strongly disagree, disagree, neutral, agree, strongly agree
The act of placing the ECG leads in the immersive setting gave me a better idea of "how" ECG leads are placed in a clinical setting.	Strongly disagree, disagree, neutral, agree, strongly agree
The act of placing the ECG leads in the immersive setting gave me a better idea of "where" each lead is placed in a clinical setting (i.e., intercostal spaces (ICS) and limbs).	Strongly disagree, disagree, neutral, agree, strongly agree
The VR experience enhanced my understanding of 12-lead ECG placement.	Strongly disagree, disagree, neutral, agree, strongly agree
The VR experience allowed me to practice and effectively apply 12-lead ECG placement techniques.	Strongly disagree, disagree, neutral, agree, strongly agree
I felt actively engaged in the learning process during the VR experience.	Strongly disagree, disagree, neutral, agree, strongly agree
The kinesthetic/tactile aspects of the VR experience helped me grasp the concept of 12-lead ECG placement better.	Strongly disagree, disagree, neutral, agree, strongly agree
I believe that VR is an effective learning tool for students with my learning style.	Strongly disagree, disagree, neutral, agree, strongly agree
The VR experience catered well to my preferred learning style.	Strongly disagree, disagree, neutral, agree, strongly agree
The VR experience made it easier for me to remember and recall 12-lead ECG placement techniques.	Strongly disagree, disagree, neutral, agree, strongly agree
I found it easier to understand the spatial relationships of ECG leads using VR than traditional learning methods.	Strongly disagree, disagree, neutral, agree, strongly agree
The VR experience improved my confidence in performing 12-lead ECG placement.	Strongly disagree, disagree, neutral, agree, strongly agree
I would recommend VR as a kinesthetic/tactile learning tool for 12-lead ECG placement to other students with different learning styles than mine.	Strongly disagree, disagree, neutral, agree, strongly agree

The survey questions were designed to identify whether the participants felt that the VR tool was effective for them and was something they would consider using again in the future. The results provide insights into the potential utility of VR as a medical education tool. Participants were presented with various answer choices depending on the survey question, ranging from frequency scales and yes/no responses to agreement levels from strongly disagree (1) to strongly agree (5).

## Results

The results from the 75 participants of this study showed that this experience was the majority of student participants' first interaction with VR or AR technology. Of the respondents, 18.6% (N = 14) indicated they had used AR or VR more than three times in their experience. This was calculated from the data in Table 2.

**Table 2:** Virtual reality usage frequency distribution survey results

Estimated frequency	Number of responses
Never	33
1-2 times	28
3-4 times	4
6-10 times	3
10+ times	7

**Figure 2:**
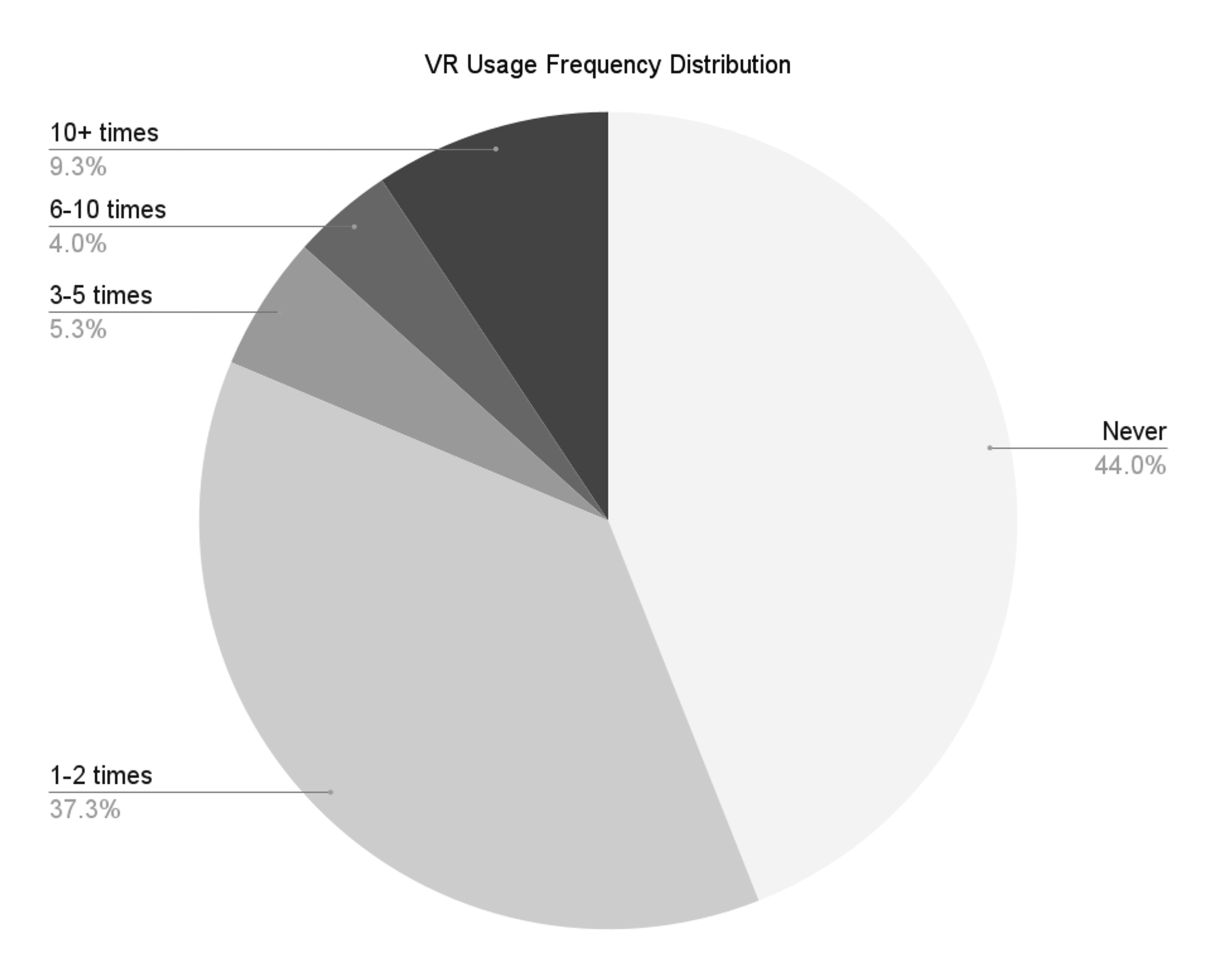
Virtual reality usage frequency distribution survey results-pie chart VR: virtual reality

This group of 14 participants comprises those who have used VR anywhere from three to 10 or more times. To illustrate, Figure 2 shows that 5.3% of participants (N = 4) reported three to five exposures to VR or AR, 4.0% of participants (N = 3) have used VR about six to 10 times, and 9.3% of participants (N = 7) have used VR more than 10 times. Of these 14 participants, 14.7% used it in educational content, 38.7% used it for entertainment, and 5.3% reported using it for both, as seen in Table 3.

**Table 3:** Previous virtual reality or augmented reality usage and preference survey results VR: virtual reality; AR: augmented reality; %: percentage

VR/AR usage category	Percentage of participants (%)	Sample variance	Population variance	Standard deviation
I've used VR or AR for EDUCATION in the past (yes or no)	14.7	0.13	0.13	0.36
I've used VR or AR for ENTERTAINMENT in the past (yes or no)	38.7	0.24	0.24	0.49
I've used VR or AR for BOTH in the past (yes or no)	5.3	0.05	0.05	0.23

**Figure 3:**
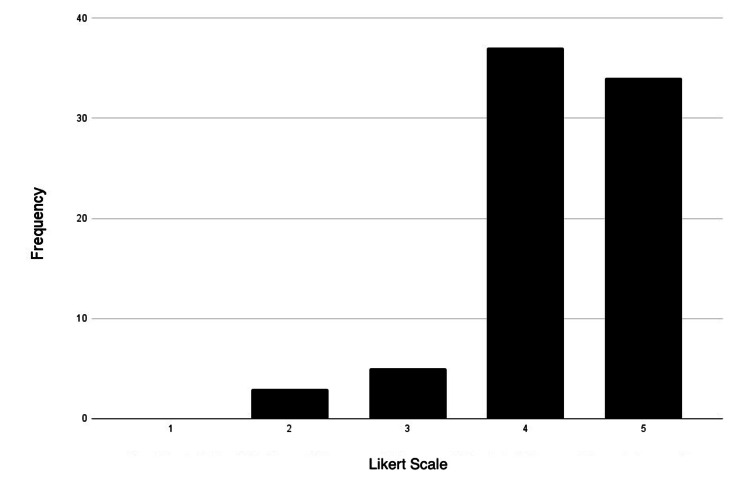
Visual learner distribution Likert scale rating (1-5): strongly disagree: 1; disagree: 2; neutral: 3; agree: 4; strongly agree: 5

**Figure 4:**
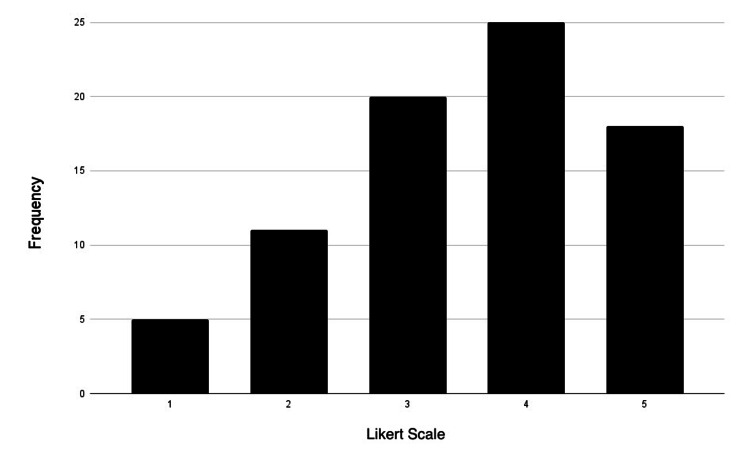
Aural (acoustic) learner distribution Likert scale rating (1-5): strongly disagree: 1; disagree: 2; neutral: 3; agree: 4; strongly agree: 5

**Figure 5:**
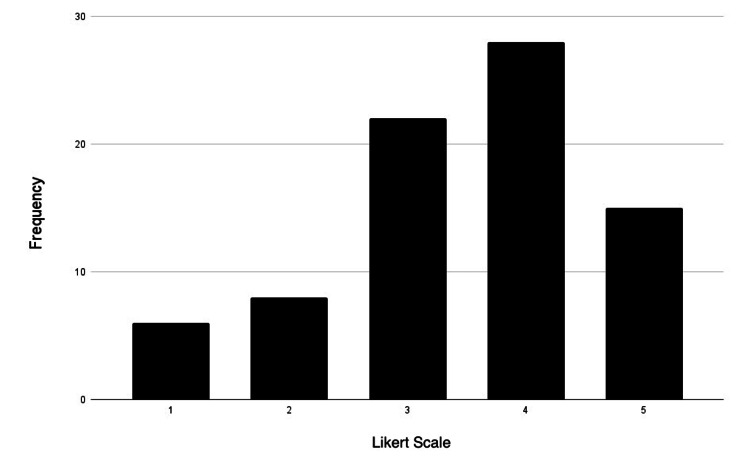
Reading/writing learner distribution Likert scale rating (1-5): strongly disagree: 1; disagree: 2; neutral: 3; agree: 4; strongly agree: 5

**Figure 6:**
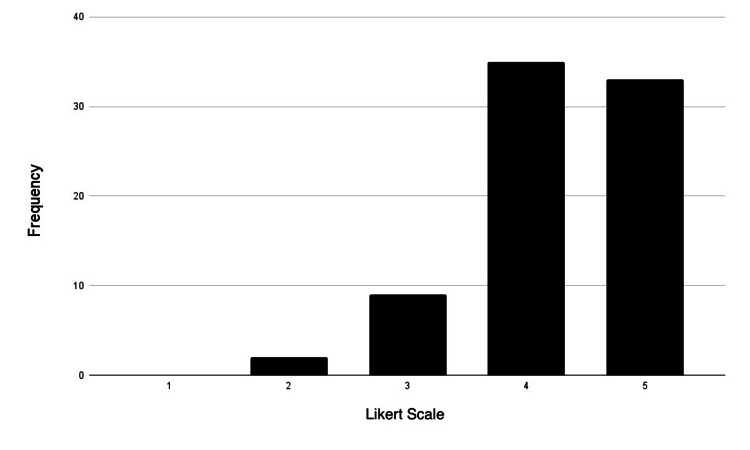
Kinesthetic learner distribution Likert scale rating (1-5): strongly disagree: 1; disagree: 2; neutral: 3; agree: 4; strongly agree: 5

Most participants identified themselves as visual and kinesthetic learners, as 90% of participants either agreed or strongly agreed with being visual learners (34 agreed; 33 strongly agreed). Additionally, 73% of participants agreed or were strongly inclined to be kinesthetic learners (32 agreed; 32 strongly agreed). Interestingly, the smallest group of participants identified as reading/writing learners (25 agreed; 14 strongly agreed). Figures 3-6 represent the frequency distribution of each learning style, based on the data from Table 4.

**Table 4:** Preferred learning style-frequency distribution

Learning style	Strongly disagree	Disagree	Neutral	Agree	Strongly agree
Visual	0	3	5	34	33
Aural	5	8	19	25	18
Reading/writing	6	8	22	25	14
Kinesthetic	0	2	9	32	32

Analysis of VR tool effectiveness

The following results are the calculated means score from the Likert scale rating for 13 of the statements from the survey questions listed in Table 1 from the methods section. The 13 statements have been organized into six main categories to better analyze the data and to allow for a more focused evaluation of the results. The 13 statements that fall within the categories can be found in Table 5.

**Table 5:** Categories and Likert-scale assessed statements on VR use in ECG lead placement VR: virtual reality; NSU-KPCOM: Nova Southeastern University Dr. Kiran C. Patel College of Osteopathic Medicine; ECG: electrocardiogram

Category	Statements
General Perception of VR Tools	Statement 1: Supplemental VR tools would have been beneficial in the NSU-KPCOM’s Cardiology course.
Understanding and Application of ECG Lead Placement	Statement 2: The act of placing the ECG leads in the immersive setting gave me a better idea of "how" ECG leads are placed in a clinical setting.
	Statement 3: The act of placing the ECG leads in the immersive setting gave me a better idea of "where" each lead is placed in a clinical setting (i.e., intercostal spaces (ICS) and limbs).
	Statement 4: The VR experience enhanced my understanding of 12-lead ECG placement.
	Statement 5: The VR experience allowed me to practice and effectively apply 12-lead ECG placement techniques.
Engagement and Learning Process	Statement 6: I felt actively engaged in the learning process during the VR experience.
	Statement 7: The kinesthetic/tactile aspects of the VR experience helped me grasp the concept of 12-lead ECG placement better.
Alignment With Learning Styles	Statement 8: I believe that VR is an effective learning tool for students with my learning style.
	Statement 9: The VR experience catered well to my preferred learning style.
Memory and Recall	Statement 10: The VR experience made it easier for me to remember and recall 12-lead ECG placement techniques.
	Statement 11: I found it easier to understand the spatial relationships of ECG leads using VR than traditional learning methods.
Confidence and Recommendation	Statement 12: The VR experience improved my confidence in performing 12-lead ECG placement.
	Statement 13: I would recommend VR as a kinesthetic/tactile learning tool for 12-lead ECG placement to other students with different learning styles than mine.

The 75 participants in this study responded to each of the 13 statements by selecting a value ranging from 1 to 5 on a Likert scale, with strongly agree = 5, agree = 4, neutral = 3, disagree = 2, and strongly disagree = 1.

**Figure 7:**
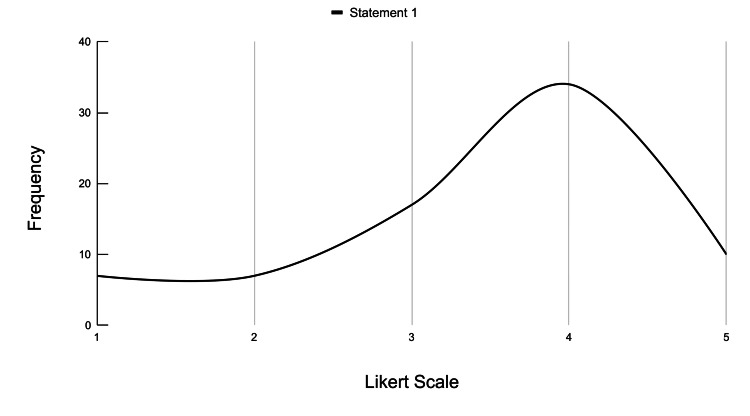
Frequency distribution of Likert scale responses for general perception of VR tools across statement 1 Likert scale rating (1-5): strongly disagree: 1; disagree: 2; neutral: 3; agree: 4; strongly agree: 5 VR: virtual reality

Statement 1 assessed the participants' general perceptions of the VR experience as it related to the benefit they believe it could have provided to their understanding of their previous cardiology coursework. The results were as follows: 10 participants strongly agreed, 34 participants agreed, 17 participants were neutral, seven participants disagreed, and seven participants strongly disagreed with statement 1, which stated that supplemental VR tools would have been beneficial in NSU-KPCOM's Cardiology course. The mean score for this statement was 3.44, indicating that most participants either strongly agreed or agreed with the statement. This data is represented in Figure 7.

Statements 2-5 assess how this particular 12-lead ECG placement VR experience affected the survey participants' understanding of the application of ECG lead placement in a clinical setting. Regarding statement 2, 13 participants strongly agreed, 29 participants agreed, 17 were neutral, nine participants disagreed, and seven participants strongly disagreed that the act of placing the ECG leads in the immersive setting gave them a better idea of "how" ECG leads are placed in a clinical setting (mean score = 3.43). In regard to statement 3, 13 participants strongly agreed, 29 participants agreed, 16 were neutral, seven participants disagreed, and 10 participants strongly disagreed with the idea that placing the ECG leads in the immersive setting gave them a better understanding of "where" each lead is placed (mean score = 3.37). For statement 4, 16 participants strongly agreed, 34 participants agreed, 12 were neutral, six participants disagreed, and seven participants strongly disagreed with the assertion that the VR experience enhanced their understanding of 12-lead ECG placement (mean score = 3.61). Finally, for statement 5, 19 participants strongly agreed, 30 participants agreed, 17 were neutral, five participants disagreed, and seven participants strongly disagreed with the statement that the VR experience allowed them to practice and effectively apply 12-lead ECG placement techniques (mean score = 3.65).

**Figure 8:**
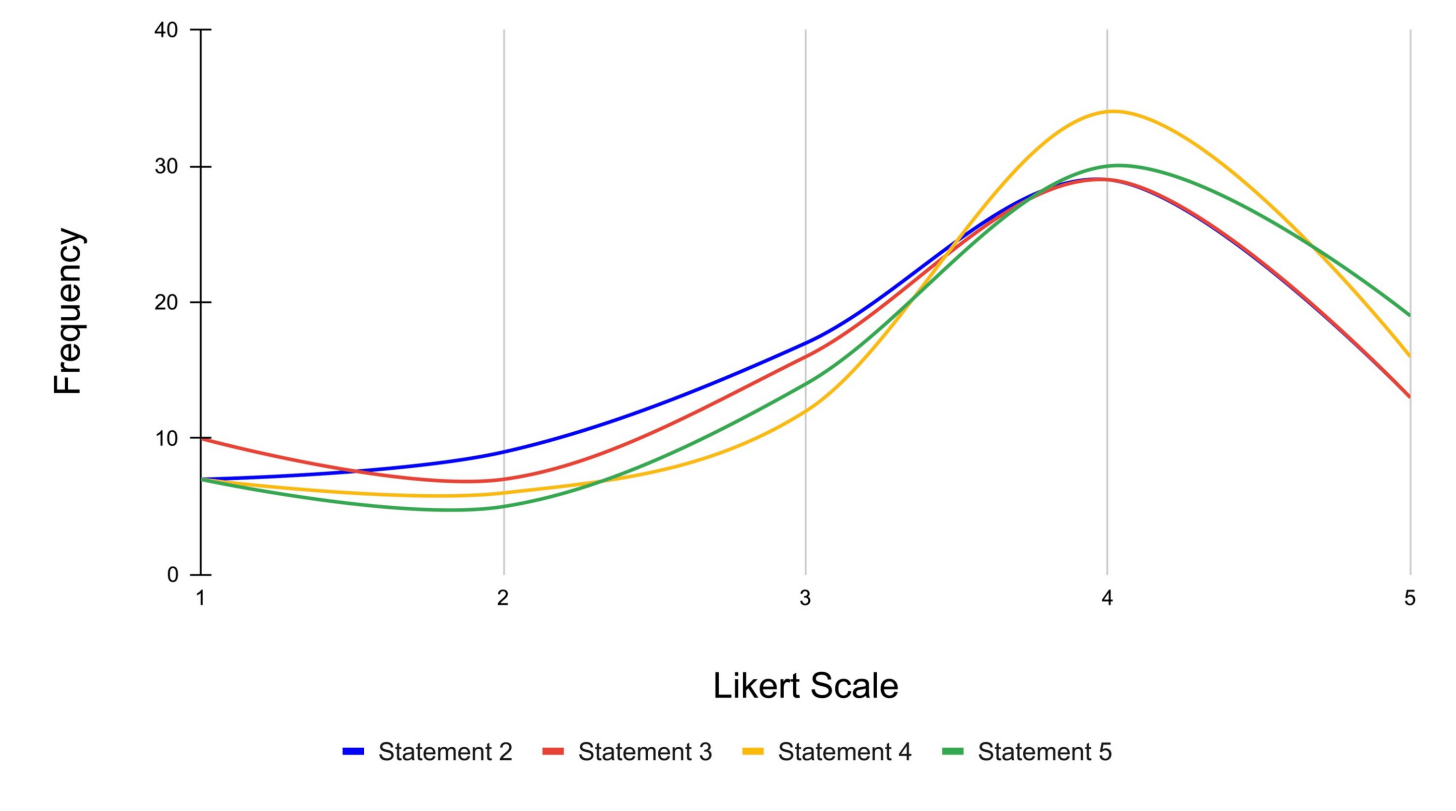
Frequency distribution of Likert scale responses for understanding and applications of ECG lead placement across statements 2 through 5 Likert scale rating (1-5): strongly disagree: 1; disagree: 2; neutral: 3; agree: 4; strongly agree: 5 ECG: electrocardiogram

Overall, the majority of students either agreed or strongly agreed with statements 2-5, further reflecting a generally positive consensus that VR is a useful academic tool for helping medical students learn clinical skills. It is important to point out that while the majority of participants did agree with these statements, the averages for statements 2 and 3 were relatively lower than the averages of the other statements proposed in this survey. This suggests that there may be areas of improvement regarding the haptic quality of VR. For example, because the nature of VR in its current stage of development does not allow participants to physically feel their virtual patient, or appreciate the amount of force and pressure required to apply a lead in real life, this limits how well the VR experience can truly mimic reality and teach the student to understand “how” and “where” an ECG lead would be placed in a real-life clinical setting. The frequency distribution for statements 2-5 is represented in Figure 8.

Statements 6 and 7 assess how actively engaged the participants felt with the learning process and how the kinesthetic aspects of the VR experience augmented the learning process, respectively. For statement 6, 20 participants strongly agreed, 32 participants agreed, 13 were neutral, six participants disagreed, and four participants strongly disagreed with statement 6, which stated that they felt actively engaged in the learning process during the VR experience (mean score = 3.77). For statement 7, 21 participants strongly agreed, 30 participants agreed, 13 were neutral, four participants disagreed, and seven participants strongly disagreed with the idea that the kinesthetic aspects of the VR experience helped them grasp the concept of 12-lead ECG placement better (mean score = 3.72).

**Figure 9:**
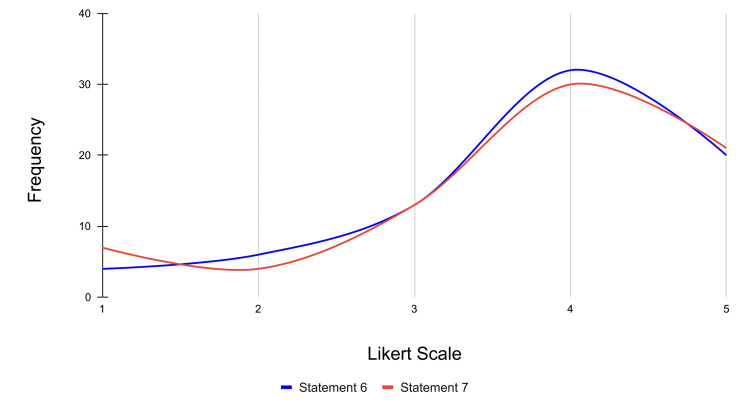
Frequency distribution of Likert scale responses for engagement and learning process across statements 6 and 7 Likert scale rating (1-5): strongly disagree: 1; disagree: 2; neutral: 3; agree: 4; strongly agree: 5

It is important to note that these two statements had the highest mean relative to the other statements in the survey. This shows that participants found VR to be engaging and that the kinesthetic quality of the VR experience was something that the students felt enhanced their learning. It is possible that the VR simulation was engaging to the participants because of the kinesthetic component, which required them to physically move within the simulation and interact with the virtual imagery. As more than half of the participants identified as being kinesthetic learners, the kinesthetic components of VR likely resonated well with them because of a preference for interactive and immersive learning tools over traditional classroom learning, which is often less interactive. The frequency distribution for statements 6 and 7 is represented in Figure 9.

Statement 8 assesses participants' belief that VR is an effective learning tool for students with their preferred learning styles. Specifically, 19 participants strongly agreed, 32 participants agreed, 13 were neutral, four participants disagreed, and seven participants strongly disagreed with this statement (mean score = 3.70). Statement 9 evaluates how well the VR experience catered to participants' specific learning styles. For this statement, 18 participants strongly agreed, 26 participants agreed, 17 were neutral, five participants disagreed, and nine participants strongly disagreed (mean score = 3.51).

**Figure 10:**
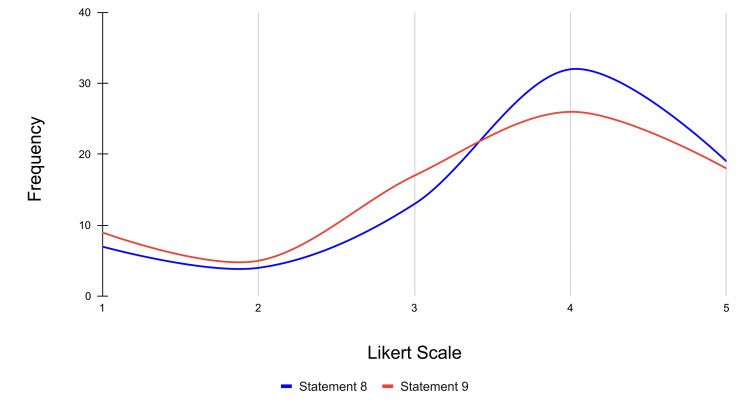
Frequency distribution of Likert scale responses for alignment with learning styles across statements 8 and 9 Likert scale rating (1-5): strongly disagree: 1; disagree: 2; neutral: 3; agree: 4; strongly agree: 5

Both statements received a majority positive agreement and reflect that VR is useful for a broad range of learning styles, particularly for those who are visual or kinesthetic learners. The frequency distribution for statements 8 and 9 is represented in Figure 10.

Statement 10 evaluates whether the VR experience improved participants' ability to remember and recall 12-lead ECG placement techniques. Specifically, 16 participants strongly agreed, 28 participants agreed, 14 were neutral, 10 participants disagreed, and seven participants strongly disagreed with the statement (mean score = 3.48). Statement 11 examines whether VR made it easier for participants to understand the spatial relationships of ECG leads compared to traditional learning methods. For this statement, 20 participants strongly agreed, 24 participants agreed, 16 were neutral, eight participants disagreed, and seven participants strongly disagreed (mean score = 3.56).

**Figure 11:**
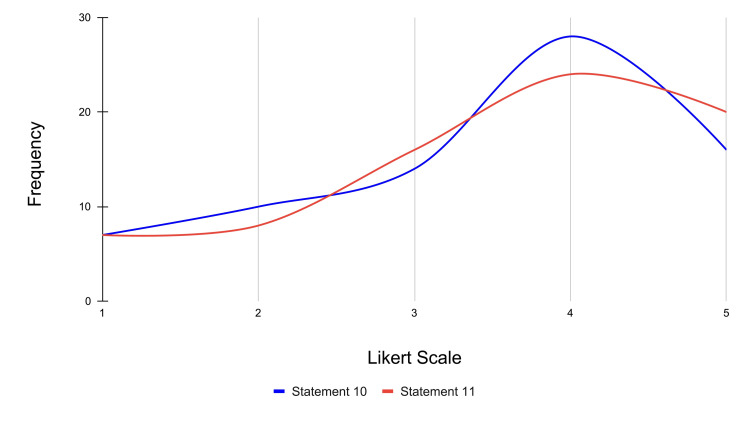
Frequency distribution of Likert scale responses for memory and recall across statements 10 and 11 Likert scale rating (1-5): strongly disagree: 1; disagree: 2; neutral: 3; agree: 4; strongly agree: 5

Overall, both statements had a majority positive agreement. The frequency distribution for statements 10 and 11 is represented in Figure 11.

**Figure 12:**
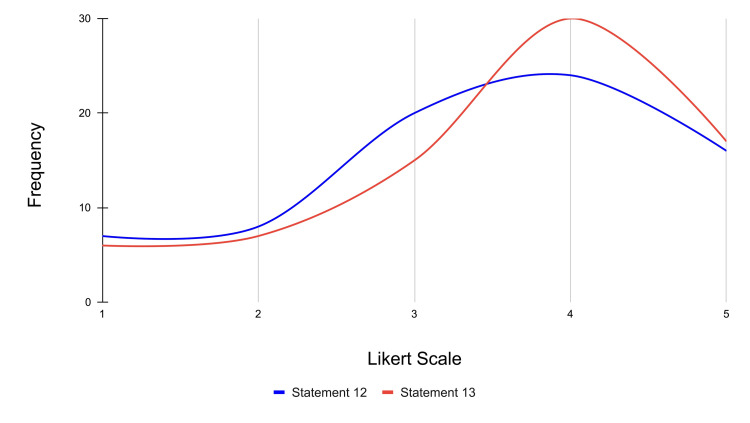
Frequency distribution of Likert scale responses for confidence and recommendation across statements 12 and 13 Likert scale rating (1-5): strongly disagree: 1; disagree: 2; neutral: 3; agree: 4; strongly agree: 5

Finally, statement 12 assesses whether the VR experience increased participants' confidence in performing 12-lead ECG placement. Specifically, 16 participants strongly agreed, 24 participants agreed, 20 were neutral, eight participants disagreed, and seven participants strongly disagreed with the statement (mean score = 3.47). Statement 13 evaluates participants' willingness to recommend VR as a kinesthetic/tactile learning tool for 12-lead ECG placement to students with different learning styles. For this statement, 17 participants strongly agreed, 30 participants agreed, 15 were neutral, seven participants disagreed, and six participants strongly disagreed (mean score = 3.62). The frequency distribution for statements 12 and 13 is represented in Figure 12.

## Discussion

Several studies have highlighted the effectiveness of VR in medical education. VR has proven to be a practical tool for student training to develop the skills needed for clinical practice [4,7]. VR’s immersive nature makes it beneficial for teaching subjects such as anatomy, radiology, and surgical procedures, as it allows students to visualize structures in a three-dimensional environment and interact with structures in the virtual setting [5,11]. For instance, VR allows students to interact with human body models in a controlled, standardized learning environment. It reduces the stress of making mistakes in front of patients. Moreover, students can repeatedly practice their clinical skills without facing negative consequences [2,3]. VR allows students to progress at their pace while engaging with scenarios that enhance their learning experience and retention of information [16].

This research aimed to explore student perceptions of VR in terms of learning clinical skills. The findings indicate that VR can keep users engaged through its kinesthetic and tactile qualities when placing the ECG leads in the virtual environment. This benefits students who learn kinetically, and the survey results showed that 73% of participants identified as kinesthetic learners. Overall, the responses to the survey for statements 1-13 highlight a generally positive consensus that VR is a useful academic tool for learning in medical school. The VR technology used in this study enhanced the majority of the medical students’ understanding and engagement in the 12-lead ECG placement performed for this study. These results showed that the highest-rated aspects of the VR experience were its ability to keep users engaged, as well as the kinesthetic aspects that allowed students to move in space. This suggests that VR not only serves as an effective tool for enhancing comprehension of clinical skills but also offers an interactive experience that aligns well with most medical students' learning preferences, particularly for those who enjoy learning in a hands-on, kinesthetic learning environment.

On the other hand, several scores indicated room for improvement as it pertains to using VR to develop clinical skills. For example, the statement “Placing the ECG leads in the immersive setting gave me a better idea of ‘where’ each lead is placed in a clinical setting (i.e., intercostal spaces (ICS) and limbs)” had the lowest average of 3.38. Furthermore, the statement “The act of placing the ECG leads in the immersive setting gave me a better idea of 'how' ECG leads are placed in a clinical setting” scored 3.43, the second lowest average. Considering that these averages are greater than 3.0, the value for neutral, still meant that the majority of students agreed with the statement. However, they had the fewest number of students to agree. It is possible that these areas scored relatively lower because of the physical limitation of VR. While VR is physically engaging, as it requires users to physically move within the simulation, the VR tools used in this study were limited in their ability to fully replicate the physical nature of reality such as the ability to perceive weight, texture, temperature, pressure, etc. For example, during the ECG lead immersive experience, participants were not able to physically feel the patient, palpate for bony landmarks, or get a sense of the amount of force and pressure required to pick up a lead and place it on a patient’s chest. However, these limitations do not negate the fact that the students in the study found the VR experience to still be beneficial to their learning and academic engagement. The limitations discovered through this study highlight the need for further development to enhance VR tactile technology for VR to maximize VR’s ability to fully simulate real-life clinical skills. For example, there is currently an AR tool, called Merge Cube, that provides users with tactile feedback while engaging with objects in a virtual setting. The Merge Cube allows anatomical structures to be tagged to a three-dimensional cube, allowing users to move and manipulate the cube in space; it changes the way the virtual image is perceived [17].

Based on the data collected, VR is beneficial in medical education and should be integrated into the curriculum of more medical schools due to its ability to enhance student engagement, cater to diverse learning styles, and provide a safe environment for repeated practice of clinical skills. However, while VR is useful in these areas, future research is needed to develop and incorporate more advanced haptic feedback, enabling students to better appreciate critical physical information such as weight, texture, and pressure, which are necessary to better understand nuanced clinical skills like 12-lead ECG placement.

The main limitation of this study is its sample size. This study consisted of 75 participants. All of them were first-year medical students from the same medical institution. This limitation restricts how broadly the data from this study can be generalized to a larger population. Additionally, this study relies on self-reported data from the participants. This creates the potential for biases, as the participants’ opinions could be influenced by the other variables that may have been introduced in this VR experience, such as technological difficulties, “tech-savviness,” and prior familiarity with 12-lead ECG placement. Furthermore, the study only used a single VR tool, Acadicus, which could restrict how broadly the findings can apply to other VR platforms or educational materials. Lastly, The VR technology that was used for the VR stimulation was Oculus Rift S, which is not the latest VR technology, so this study does not consider advancements that are available with more up-to-date VR tools.

## Conclusions

In conclusion, this research explored the benefits of using VR in medical school education in the context of 12-lead ECG placements. The findings indicated that most participants found that the VR tool effectively enhanced their understanding and engagement with the clinical procedure. This study recognized the positive impact that VR technology had on students' confidence levels in the clinical skill of 12-lead ECG placement. Furthermore, based on the data collected, VR was beneficial in medical education and should be integrated into the curriculum of more medical schools due to its ability to enhance student engagement, cater to diverse learning styles, and provide a safe environment for repeated practice of clinical skills. This research provides insights into how medical students perceive VR regarding learning clinical skills. Lastly, it is important to note that while virtual and AR technologies are still in their early stages of development, advancements such as haptic feedback integration and gesture tracking are necessary for making VR as reflective of reality as possible. This area of development shows promise for further enhancing immersive capabilities, and further research into the importance of a haptic response in a VR learning environment is warranted.
